# Phenotypic and genotypic detection of metallo-β-lactamases in *A*. *baumanii* isolates obtained from clinical samples in Shahrekord, southwest Iran

**DOI:** 10.1186/s13104-019-4636-y

**Published:** 2019-09-18

**Authors:** Mansoor Khaledi, Milad Shahini Shams Abadi, Majid Validi, Behnam Zamanzad, Rezvan Vafapour, Abolfazl Gholipour

**Affiliations:** Department of Microbiology and Immunology, Cellular and Molecular Research Center, Basic Health Sciences Institute, Shahrekord University of Medical Sciences, Shahrekord, Islamic Republic of Iran

**Keywords:** *Acinetobacter baumanii*, Metallo-beta-lactamase, Carbapenems, Iran

## Abstract

**Objective:**

*Acinetobacter baumanii* is a pathogenic bacterium that is the cause of many nosocomial infections. This study aimed to determine metallo-β-lactamases (MBL) produced by the *A*. *baumanii* isolates obtained from clinical samples in Shahrekord, southwest Iran.

**Results:**

A total of 100 *A*. *baumanii* were isolated from 250 clinical samples between June 2013 and June 2014. Then, the isolates were identified by biochemical tests, and MBL screening was conducted by the phenotypic tests modified Hodge, EDTA-disk synergy (EDS), combined disk (CD) and AmpC disc after antibiotic sensitivity test. Using PCR technique the bla genes were detected. Eighty-five (85%) isolates were resistant to meropenem and imipenem. Phenotypic tests showed that out of the 100 isolates, 46, 59, 50, 65 and 65 isolates were positive: AmpC disk, CD, EDS, Modified Hodge and E-test MBL respectively. Gene detection by PCR showed that 23 isolates carried the VIM-1 gene and only three isolates carried the IMP-1 gene. The prevalence of metallo-β-lactamases isolates containing *A*. *baumanii* is increasing. Furthermore, the coexistence of various carbapenemases is dominantly act as a major problem. Continuous monitoring of the infections related to these bacteria should be considered to plan an alternative and new therapeutic strategies.

## Introduction

*Acinetobacter baumanii* is recognized for human as a pathogenic bacterium that has the potential to acquire antibiotic resistance and significant inherent resistance in latest years. *A. baumanii* can survive in distinct circumstances of the environment. This bacterium is the most common pathogen that is responsible for nosocomial infections, including hospital-acquired pneumonia and urinary tract, central nervous system, skin, smooth tissue, bloodstream, bone, and surgical site infections [1]. *A. baumanii’s* antibiotic resistance is one of the reasons why such diseases spread because such bacteria can intrinsically transfer resistance factors to each other [2]. Therefore, the treatment of *A*. *baumanii* infections have recently become challenging due to acquisition of resistance to numerous antibiotics by intrinsic and acquired mechanisms [3, 4]. Carbapenems are a class of antibiotics that are effective on gram-negative and gram-positive bacteria [3, 4]. These pathogens are resistant to penicillinases and cephalosporins [5]. The main antibiotic resistance mechanism in these classes is carbapenemase synthesis [6, 7]. These carbapenems can be hydrolyzed by a class of β-lactamases categorized as B subclass by Ambler’s classification and known as metallo-β-lactamases (MBL). MBLs lead to numerous antibiotics resistance including penicillin, cephalosporins and carbapenems [5]. Decreased permeability of the outer membrane and increased efflux pump are the other mechanisms of carbapenem resistance [8, 9]. There are a number of MBLs genes such as imipenemase (IMP), Verona integron-encoded metallo-beta-lactamases (VIM), Sno Paolo metallo (SPM), New-Delhi metallo-β-lactamase (NDM), German imipenemase (GIM), Kyorin University Hospital imipenemase (KHM), and Australian imipenemase (AIM) [10, 11]. IMP, VIM and SPM are the most important genes of MBLs that have been detected in *A*. *baumanii* [12]. It has been suggested that different phenotypic tests identify MBLs based on metal-chelating ability such as EDTA inhibiting MBL activity [13]. The phenotypic experiments were used in the present research to modify Hodge, double disk synergy (DDS), and AmpC disk tests. This research was carried out using phenotypic and genotypic methods to identify MBLs generated baumanii isolates. All the isolates obtained from clinical samples at teaching hospitals in Shahrekord, southwest Iran.

## Main text

### Materials and methods

#### Patients and sampling

Between June 2013 and May 2014, a total of 250 clinical samples including trachea, blood, urine, wound culture, cerebrospinal fluid, and pleural effusion was collected from the inpatients in different wards of Kashani and Hajar Hospitals of Shahrekord. This project was approved by Shahrekord University of Medical Sciences Ethics Committee, and all informed participants complemented a written consent.

#### Isolation and detection of *A. baumanii* isolates

*A*. *baumanii* strains were isolated and identified by common chemical tests and techniques [14].

#### Antimicrobial susceptibility test

Antimicrobial susceptibility test was conducted by Kirby-Bauer protocol (disk diffusion) using 10 µg imipenem and meropenem discs, E-test strips of these antibiotics, and MBL strip. Zone inhibition diameters were measured, and the results were interpreted with reference to the Clinical & Laboratory Standards Institute (CLSI) (CLSI 2013). *Pseudomonas aeruginosa* (ATCC^®^ 2785) was used as qualitative control.

#### Modified Hodge test

**Fig. 1:**
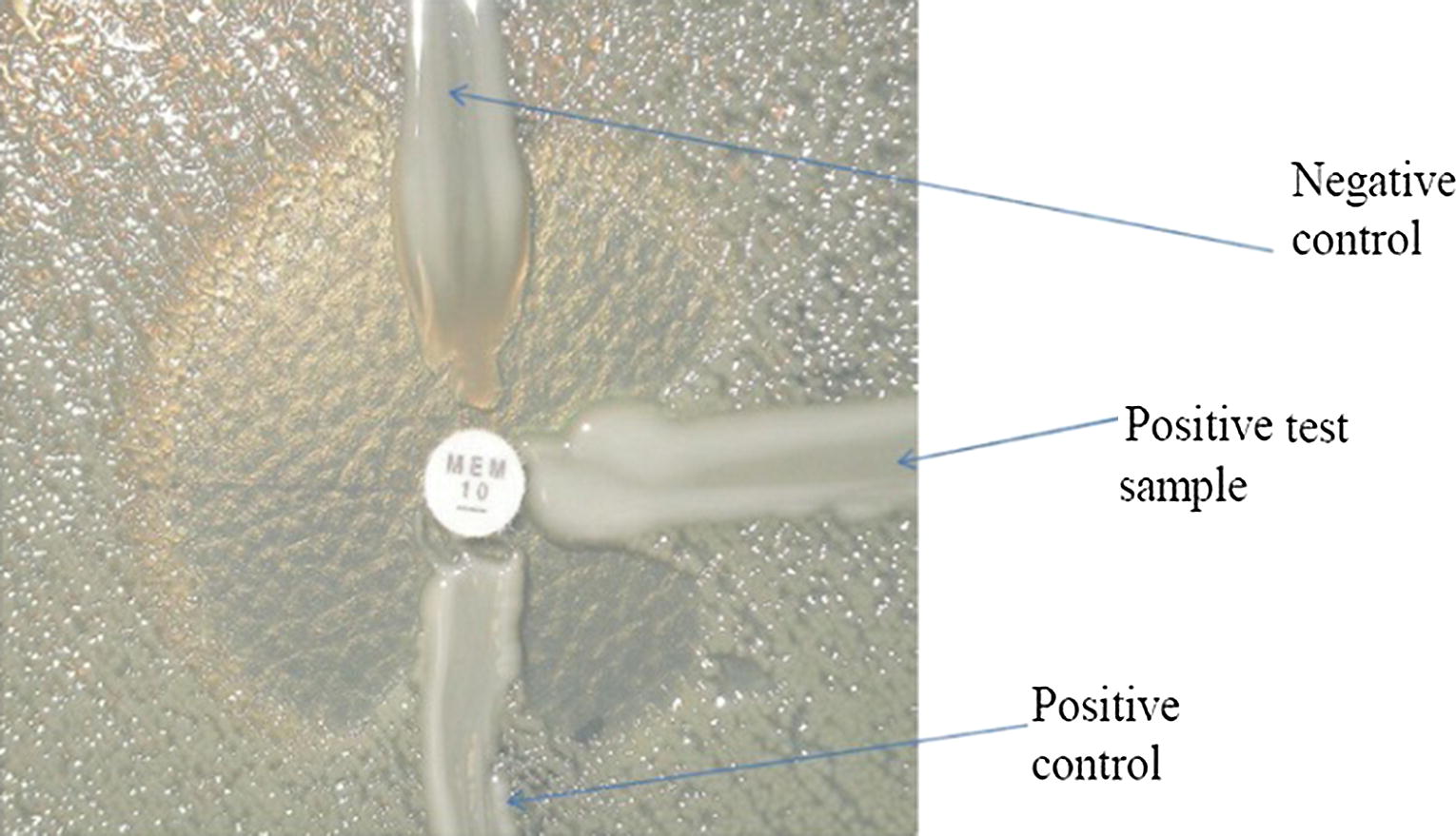
Modified Hodge test. Positive strain shows a ‘cloverleaf shaped’ zone of inhibition due to carbapenemase production, while the negative strain shows an undistorted zone of inhibition

A Modified Hodge test was conducted to screen for carbapenemase [15, 16]. After suspension culture of *Escherichia coli* (ATCC 25922), a carbapenem-susceptible organism, for 24 h to reach a bacterial turbidity of 0.5 McFarland standard (NCCLS 2000). The bacteria were cultured in the Mueller–Hinton agar using a swab, and then the 10 µg meropenem disk was placed in the center of the plate. After a 24-h culture, the bacteria did not grow around the disc. After a 24-h incubation at 37 °C, the existence of inhibition zone was considered positive control (Fig. 1).

#### EDS and CD tests

EDS test was performed on meropenem- and imipenem-resistant isolates using meropenem and ceftazidime, and CD test to detect MBLs [16, 17]. Practically, a bacterial suspension of the 0.5 McFarland standard was cultured on the Mueller–Hinton agar, and then two 10 µg carbapenem (IMI and MEM) discs as well as a 30 µg ceftazidime disc were added to the Mueller–Hinton agar. Subsequently, the MBL stop solution (10 µl) was added to the culture medium. After a 24-h incubation at 35 °C, the inhibition zone diameter was measured and compared with other discs. For the EDS test, a bacterial suspension of the 0.5 McFarland standard was prepared from the isolates, and the samples were cultured in Mueller–Hinton agar. A 10 µg imeropenem disc was placed in the agar medium. A blank disc was kept in the inner surface of the Mueller–Hinton agar and 10 µl of 0.5 M EDTA was added. Likewise, the EDTA disc was transferred to the agar surface and kept at 10 mm distance from the meropenem or ceftazidime disc. After a 24-h incubation at 37 °C, the presence of inhibition zone between the two discs was considered to represent MBL production.

#### AmpC disk test

AmpC disk test was conducted to detect MBL in imoperem-resistant strains [17–19].

#### DNA extraction

The DNAs of the bacterial isolates were extracted using a DNA extraction kit (Bioneer, Korea, Cat. No. K-3032-2-).

#### Detection of bla (IMP) and bla (VIM) using PCR

PCR was performed to screen for the VIM and IMP genes. Table 1 shows the used primers. PCR consisted of forward and reverse primers, DNA pattern, and Master Mix. The PCR product was analyzed by polyacrylamide gel.

**Table 1:** PCR primers used in this study

Primers	Primer sequence	Product size	References
VIM-1 F VIM-1 R	TGGTTGTATACGTCCCGTCA:F TGTGTGCTGGAGCAAGTCTA R:	206 bp	This study
IMP-1F IMP-1R	TAACGGGTGGGGCGTTGTTCCT:F CGCCCGTGCTGTCGCTATGAAA R:	bp 179	This study
OXA-51 F OXA-51 R	TAATGCTTTGATCGGCCTTG:F TGGATTGCACTTCATCTTGG R:	bp 353	Hu et al. [26]

#### Data analysis

Data including questionnaire data and the results of phenotypic and genotypic tests were analyzed by Chi square and Fisher’s exact test in the SPSS version 22.

## Results

Eighty-five (85%) samples were resistant to meropenem and imipenem. 43 number of patients (43%) were female. The mean age of the participants was 47.2 years (range; 1 dy-88 years). The minor and major prevalence of isolated bacteria were allocated to the tracheal and abscess samples respectively.

The tracheal samples carried the most bacteria containing the MBLs. Fisher’s exact test showed that the associations between the samples and the presence of bla VIM-1 (*p *= 0.28) and bla IMP-1 (*p *= 0.88) were not statistically significant.

### Resistance to meropenem and imipenem

After incubation of the plates containing the meropenem and imipenem discs and E-test strips, the diameters of the inhibition zones were measured and interpreted with reference to the CLSI. For the E-test strips, minimum inhibitory concentration (MIC) was observed in the areas where inhibition zone diameters was increased. E-test with (MIC > 32) indicated that 85 samples of the 100 *A*. *baumanii* isolates were resistant to meropenem and imipenem (carbapenem). Based on disk diffusion assay, 81 samples were resistant and four samples relatively resistant (semi-susceptible).

### The results of phenotypic tests for detecting MBLs

46 (46%) cases out of 100 strains were AmpC beta-lactamase producers, and 59 strains positive by CD test, representing MBL production. In addition, 50 samples were found to be positive in E-test (EDTA disk synergy test). Sixty-five samples were found as carbapenemase producing by the modified Hodge test and 65 as MBL producing by E-test.

### The results of genotypic tests

After the electrophoresis of PCR products, the bands were carefully examined. The presence of the OXA-51 gene (the specific internal gene of *A*. *baumanii*) was investigated to confirm the phenotypic tests for *A*. *baumanii* detection.

All samples carried the OXA-51 gene. The presence of this gene confirmed the results of biochemical tests for *A*. *baumanii* detection. The PCR results also showed that the *A*. *baumanii* isolates from our patients carried the VIM-1 and IMP-1 MBL genes. Twenty-three samples carried the VIM-1 gene and only three samples had the IMP-1 gene (Table 2).

**Table 2:** The VIM-1 and IMP-1 genes-producing isolates among meropenem- and imipenem-resistant *Acinetobacter baumanii* strains

VIM-1		IMP-1		Total
Positive	Negative	Positive	Negative	100
23 (23%)	77 (77%)	3 (3%)	97 (97%)	

### The association between meropenem resistance and the presence of bla IMP-1 and bla VIM-1 genes in *Acinetobacter baumanii* strains

Chi square test (α = 0.5) showed a significant association between meropenem resistance and the presence of bla VIM-1 gene in *A*. *baumanii* strains (*p *= 0.22); however, there was not relation between meropenem resistance and the presence of bla IMP-1 (*p *= 0.46).

## Discussion

*A*. *baumanii*, a bacterium with multidrug resistance, is considered a highly important pathogen that can endanger human health. This organism is the cause of numerous infections in human, and appearances mainly in the people with immunodeficiency or underlying disease. The infections due to *A*. *baumanii* is treated with broad-spectrum antibiotics [9]. However, the antibiotic resistance of this microorganism has potentially been increased, like carbapenem resistance is spreading across the world [20]. Carbapenems including imipenem and meropenem are drugs of choice for *A*. *baumanii* infection. Recently, the emergence of MBLs (carbapenemase) belonging to the classes D and B of beta-lactamases has led to the hydrolysis of these drugs, and therefore, *A*. *baumanii* gain resistance to them [21, 22]. It is highly useful to *early* detection of these genes in carbapenem resistant bacteria to control and prevent the spread of these bacteria in hospitals. These data can also help the physicians to make appropriate prognosis and prescribe suitable antibiotic regimens [5]. In this study, we investigated the prevalence of the most important MBL genes that have been identified in *A*. *baumanii* strains.

We observed that 85 (85%) isolates were resistant to meropenem and imipenem, and the prevalence of the IMP-1 and VIM-1 genes were derived 3% and 23% respectively.

Nouri et al. [23] studied the prevalence of MBLs in *A*. *baumanii* in Tehran with the most strains isolated from tracheal samples that is similar to our study. So, sterilization of respiratory tubes is one of the most important ways to prevent development of this infection in these tubes. In our study, the phenotypic tests AmpC disk, CD, EDS, modified Hodge, and E-Test MBL showed that 46, 59, 50, 65, and 65 isolates in order were MBL-producing, and only 23 isolates carried the VIM-1 gene and three isolates had the IMP-1 gene. This inconsistency in the findings can be attributed to the presence of other MBL genes or other mechanisms such as defects of porins or decreased expression of outer membrane proteins [24].

However, results of phenotypic and genotypic tests in several investigations have also consistence with our result. For example, Shoja reported that 55% of the isolates were positive by DDS test but did not find any MBL producing gene, which is consistent with the studies of Manageiro [25] and Hu [26].

Peymani et al. observed that among 63 carbapenem-resistant *A*. *baumanii* strains, 31 (49%) strains contained MBL which 19 (61%) carried bla VIM-1 and the rest had bla IMP-1 (31%). These statistics are consistent with our study with respect to the prevalence of the VIM-1 gene but inconsistent with respect to the prevalence of IMP-1 that was higher in the study of Peymani et al. [24]. The study of Kouyama et al. [27] indicated that out of the 598 *A*. *baumanii* strains isolated from the patients in the hospitals in different cities of Japan, 4.5% were resistant to imipenem and meropenem, which is a lower prevalence rate compared to different regions. This can be attributed to the genetic and climatic conditions in that region of the world. *A*. *baumanii* resistance rate to imipenem and meropenem is heterogeneous in East Asia. It has been reported 9.26% in Korea [28], 49% in Taiwan [29], 50–52.4% in China [30], and 4.5% in Japan, possibly representing the effects of geographical differences.

Turner et al. [31] conducted a large study in Europe that showed imipenem resistance rate of *A*. *baumanii* isolates at the hospital level was 22–26%. Sung et al. [32] reported that out of the 31 carbapenem-resistant *A*. *baumanii* strains, 15 (48.4%) isolates carried the bla IMP-1 gene, which is comparatively higher than our finding. In regard to these statistics, the spread and prevalence of both genes are increasing. On the other hand, the lower prevalence of bla IMP-1 in the region studied in the current study and also the high prevalence and importance of bla VIM-1 should be paid special attention.

The coexistence of different carbapenemases is considered a serious health issue that have remarkable effects on alternative and newer therapeutic strategies, stricter measures of infection control, and new design for continuous monitoring. The continuous monitoring of the prevalence of carbapenem resistance and associated mechanisms in *A*. *baumanii* strains can help to develop appropriate therapeutic strategies against nosocomial infections.

## Limitations

The lack of investigation on others resistance mechanisms in *A. baumanii* isolates can be mentioned as one of the main limitations of the present study.

## Data Availability

All relevant data are included in the manuscript.

## Abbreviations

MBL: metallo-β-lactamases
IMP: the genes imipenemase
VIM: Verona integron-encoded metallo-beta-lactamases
SPM: Sno Paolo metallo
NDM: New-Delhi metallo-β-lactamase
GIM: German imipenemase
KHM: Kyorin University Hospital imipenemase
AIM: Australian imipenemase
